# Exploring the association between birthweight and breast cancer using summary statistics from a perspective of genetic correlation, mediation, and causality

**DOI:** 10.1186/s12967-022-03435-2

**Published:** 2022-05-14

**Authors:** Meng Zhang, Jiahao Qiao, Shuo Zhang, Ping Zeng

**Affiliations:** 1 Department of Biostatistics, School of Public Health, Xuzhou Medical University, Xuzhou, 221004 Jiangsu China; 2 Center for Medical Statistics and Data Analysis, Xuzhou Medical University, Xuzhou, 221004 Jiangsu China; 3 Key Laboratory of Human Genetics and Environmental Medicine, Xuzhou Medical University, Xuzhou, 221004 Jiangsu China; 4 Key Laboratory of Environment and Health, Xuzhou Medical University, Xuzhou, 221004 Jiangsu China

**Keywords:** Breast cancer, Birthweight, Fetal/maternal-specific effect, Summary statistics, Mendelian randomization, Genetic correlation, Mediation, Gene-based association analysis, Age of menarche, Age at menopause, Pleiotropy analysis

## Abstract

**Background:**

Previous studies demonstrated a positive relationship between birthweight and breast cancer; however, inconsistent, sometimes even controversial, observations also emerged, and the nature of such relationship remains unknown.

**Methods:**

Using summary statistics of birthweight and breast cancer, we assessed the fetal/maternal-specific genetic correlation between them via LDSC and prioritized fetal/maternal-specific pleiotropic genes through MAIUP. Relying on summary statistics we conducted Mendelian randomization (MR) to evaluate the fetal/maternal-specific origin of causal relationship between birthweight, age of menarche, age at menopause and breast cancer.

**Results:**

With summary statistics we identified a positive genetic correlation between fetal-specific birthweight and breast cancer (*r*_*g*_ = 0.123 and *P* = 0.013) as well as a negative but insignificant correlation between maternal-specific birthweight and breast cancer (*r*_*g*_ = − 0.068, *P* = 0.206); and detected 84 pleiotropic genes shared by fetal-specific birthweight and breast cancer, 49 shared by maternal-specific birthweight and breast cancer. We also revealed fetal-specific birthweight indirectly influenced breast cancer risk in adulthood via the path of age of menarche or age at menopause in terms of MR-based mediation analysis.

**Conclusion:**

This study reveals that shared genetic foundation and causal mediation commonly drive the connection between the two traits, and that fetal/maternal-specific birthweight plays substantially distinct roles in such relationship. However, our work offers little supportive evidence for the fetal origins hypothesis of breast cancer originating in utero.

**Supplementary Information:**

The online version contains supplementary material available at 10.1186/s12967-022-03435-2.

## Background

Breast cancer remains the most frequent malignant tumor that occurs in the glandular epithelium of breast [1], and accounts for approximately 12% of the total 9.6 million deaths due to cancer [2]. Since 1970s, the incidence rate of breast cancer worldwide has continued to increase; it is reported that one in eight women suffered from this type of cancer in USA [3]. Although mammary gland is not an important organ to maintain human life and breast cancer in situ is not fatal, breast cancer cells may lose characteristics of normal cells and is easy to fall off due to the loose connection among cells. Once falling off, cancer cells would spread throughout the body with blood or lymph, leading to cancer metastasis and thus endangering life [4]. Over the past few decades the treatment of breast cancer has been advanced greatly, but the overall survival is still not optimistic [1, 5]. Therefore, it is particularly important to understand the etiology, occurrence, and development of breast cancer for early prevention. Existing studies have identified a series of risk factors involved in breast cancer, including dietary habit, age at first birth, age at menarche, age at menopause, family history, excessive intake of exogenous hormones as well as genetic mutations such as *BRCA1*, *BRCA2*, and *PIK3CA* [1, 6–10].

However, these traditionally established risk factors during women’s adult life appear not to adequately interpretate the occurrence pattern of breast cancer. To advance our understanding of disease causes, the relationship between breast cancer and early growth/development, perinatal intrauterine environments has been attracted much attention since 1990s [11–23]. Among those, the association between birthweight and breast cancer has also received much research interest. Although a positive correlation between women’s birthweight and breast cancer risk was discovered in studies [11, 12, 20, 23–32], some others failed to replicate such connection or even detected inconsistent correlations in effect direction [13, 15, 21, 22, 33–41]. These discrepant findings may be partly due to potential confounding influences commonly arisen in observational studies, making it difficult to draw a definitive conclusion on the causal association between birthweight and breast cancer. In addition, it is not clear whether there exists a mediating association between the two traits [20, 42].

Furthermore, from a genetic perspective, it is also not known whether the observed co-existence of low/high birthweight and breast cancer is partly driven by causal association or shared genetic background between them. Moreover, all prior studies cannot distinguish the maternal-specific and fetal-specific effects of birthweight on breast cancer from each other. Compared to other factors, birthweight is a special exposure proxy genetically affected by both mother’s and offspring’s genotypes [43]. Therefore, partitioning the overall effect of birthweight into maternal-specific and fetal-specific components holds the key for understanding the origin of the association between birthweight and breast cancer. Although the longitudinal cohort study can provide empirical evidence for causal inference, it requires large-scale populations and long-term follow-up before the onset of breast cancer [35]; consequently, the implementation is not easy. In the traditional scenario, randomized controlled trial is the gold standard for inferring causality, but such study is also infeasible to investigate the causal association between birthweight and breast cancer [29]. In addition, both the two types of studies cannot resolve the maternal-specific and fetal-specific impacts of birthweight on adult diseases including breast cancer.

**Fig. 1:**
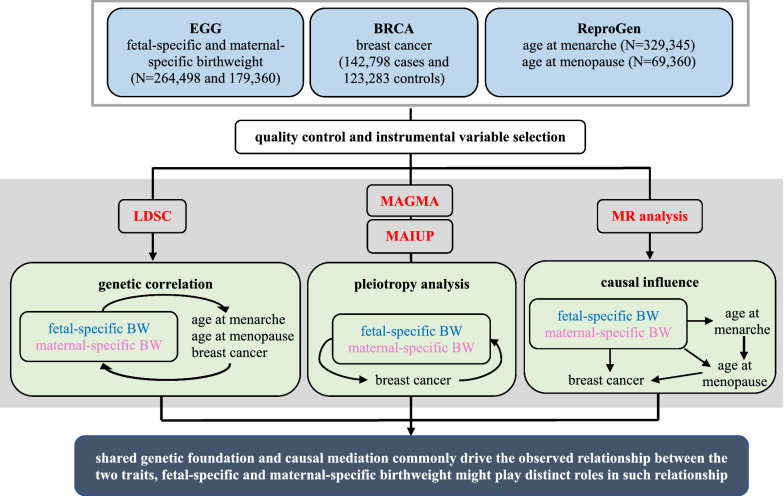
Flow diagram of data process and statistical analysis for the present study. quality control: exclude SNPs having no rs label and remove duplicated SNPs; LDSC: the cross-trait linkage disequilibrium score regression; MAGMA: Multi-marker Analysis of GenoMic Annotation; MAIUP: Mixture Adjusted Intersect-Union Pleiotropy test; MR: Mendelian randomization; BW: birthweight

The present work attempted to answer these critical questions via genetic analysis using summary-level data available from large-scale genome-wide association studies (GWASs). First, to assess the extent of genetic overlap shared between birthweight and breast cancer, we applied the cross-trait linkage disequilibrium score regression (LDSC) to quantify the genetic correlation between them [44]. Second, we employed a novel pleiotropy test method called MAIUP (Mixture Adjusted Intersect-Union Pleiotropy test) to determine pleiotropic genes [45–47]. Third, to elaborate the causal association between birthweight and breast cancer, we resorted to apply Mendelian randomization (MR) methods [48–51]. In the MR analysis, genetic variants, which are required to be associated with the exposure of focus, are used as instrumental variables, based on which the causal association between the exposure (e.g., birthweight) and the disease (e.g., breast cancer) can be inferred. Recently, one such MR study was performed but found no evidence supporting the causal association between birthweight and breast cancer [52]. However, that study did not explore the separate maternal-specific and fetal-specific effects of birthweight on breast cancer. The summary statistics of maternal/fetal-specific effects of SNPs (single nucleotide polymorphisms) on birthweight, released by a recent GWAS [43], offers us an unprecedented opportunity to untangle the maternal and fetal contributions of birthweight to breast cancer by using novel MR methods. Furthermore, as a byproduct of our MR analysis, we can evaluate the mediating relationship between birthweight and breast cancer, with age of menarche and age at menopause as two candidate mediators. The flow diagram of data process and statistical analysis for the present study is illustrated in Fig. 1.

## Methods

### GWAS summary statistics

We obtained fetal-specific and maternal-specific summary statistics (e.g., marginal effect size and standard error for SNPs) of birthweight (*n* = 264,498 for own birthweight and *n* = 179,360 for offspring birthweight) from the largest GWAS to date published by the Early Growth Genetics consortium [43]. These fetal/maternal-specific datasets provide us an in-depth understanding of biological regulation of birthweight and allows us to further investigate the origin of observed relationship between birthweight and breast cancer. We yielded summary statistics of breast cancer from [52] (*n* = 266,081), age at menarche (*n* = 329,345) [53] and age at menopause (*n* = 69,360) [54] from the ReproGen consortium. All the individuals analyzed in these studies were of European ancestry.

### Genetic correlation estimation with LDSC

To assess the shared polygenic component between fetal/maternal-specific birthweight and breast cancer, we performed LDSC to estimate the overall genetic correlation [44]. In brief, the LDSC analysis proceeded by regressing the product of Z-statistics of the two traits on the LD score in a weighted manner via the python script offered by the developers with default parameter settings. The regression slope of LDSC provided an unbiased estimate for genetic correlation even when overlapping individuals existed between the two GWASs. Before the analysis, the stringent quality control (e.g., removing SNPs located within the MHC region) was carried out on summary statistics of birthweight and breast cancer following prior work [44].

### Pleiotropic gene identification with MAIUP

Using summary statistics of birthweight and breast cancer, we attempted to further identify fetal/maternal-specific gene-level pleiotropic associations shared by the two traits. Here, pleiotropy is defined the phenomenon that a given gene is associated with both traits under investigation [55–58]. Statistically, the presence of pleiotropy means that both the *P* values (say *P*_1_ and *P*_2_) of a particular gene should be equal or less than the preassigned significance level (say *α*); that is, *P*_1_ ≤ *α* and *P*_2_ ≤ *α* (*H*_11_) need to be held simultaneously for pleiotropic association. In contrast, the absence of pleiotropy implies that at least one of the two *P* values would be larger than the significance level, which includes three sub-null scenarios; that is, (i) *H*_00_: *P*_1_ > *α* and *P*_2_ > *α*, (ii) *H*_01_: *P*_1_ > *α* and *P*_2_ < *α*, and (iii) *H*_10_: *P*_1_ < *α* and *P*_2_ > *α*. From a statistical perspective, it is easy to see that the pleiotropy detection can be viewed as a high-dimensional challenging issue of composite null hypothesis testing [45]. To address this problem effectively, we employed a recently proposed pleiotropy test method called MAIUP to detect common genetic loci underlying birthweight and breast cancer [59]. Methodologically, MAIUP is constructed under the principle of intersect-union test originally proposed within the framework of high-dimensional mediation analysis [46, 47, 59], which takes two sets of *P* values for each gene as input with a three-component mixture null distribution for its test statistics and generally behaves much better in power compared to other existing pleiotropy test methods. Technical details regarding MAIUP can be found in [59].

To generate *P* value for each gene in GWAS, we need to first integrate multiple SNP-level association signals into a single gene-level association signal. For this aim, we applied MAGMA (Multi-marker Analysis of GenoMic Annotation) which is a powerful SNP-set test method and can be efficiently conducted via user-friendly software [60]. Due to population stratification, family structures, and cryptic relatedness [61–63], the empirical null distribution in MAGMA may be sometimes inflated. In order to correct such deviation, before the formal pleiotropy analysis we performed genomic control if the inflation was observed, which was measured by the genomic control inflation factor (> 1.05) for chi-square statistics [64, 65]. Afterwards, *P* values for all genes were available for each trait. Depending on these *P* values, we conducted MAIUP to discover significant genes that were simultaneously associated with birthweight and breast cancer.

### Mendelian randomization for causal association between birthweight, age at menarche, age at menopause, and breast cancer

We finally evaluated the causal association among the four traits using various MR methods. Following prior studies [43, 66, 67], we selected a set of independent birthweight-associated SNPs (*P* < 6.60 × 10^–9^ and *r*^2^ < 0.10) as instruments. Specifically, the total of 104 instruments for fetal-specific birthweight included 63 fetal-effect specific SNPs, 26 SNPs exerting both fetal and maternal effects with the same effect direction and 15 SNPs exhibiting both fetal and maternal effects but with the opposite effect direction (Additional file 1: Table S1); while the total of 72 instruments for maternal-specific birthweight included 31 maternal-effect specific SNPs, 26 SNPs exerting both fetal and maternal effects with the same effect direction, 15 SNPs exhibiting both fetal and maternal effects but with the opposite effect direction (Additional file 1: Table S2). To avoid weak instrument bias, 71 SNPs with unclassified effect direction were excluded as fetal-specific or maternal-specific birthweight instruments. With these instruments of birthweight, we estimated the fetal/maternal-specific causal effect of birthweight on age at menarche, age at menopause, or breast cancer. In addition, to estimate the causal effect of age at menarche or age at menopause on breast cancer, we selected independent associated SNPs for age at menarche or age at menopause as candidate instruments by applying the clumping procedure in PLINK [68]. We set the primary and secondary significance levels of indexed SNPs to 5 × 10^–8^, *r*^2^ to 0.001 and physical distance to 1 Mb, with the 1000 Genomes Project as the reference panel. We estimated the causal effect primarily using the IVW method [49, 51]. To assess the robustness and credibility of our MR results, we also performed several sensitivity analyses when necessarily: (1) MR-Egger regression to evaluate the directional pleiotropy of instruments [69]; (2) weighted median-based method [70] when instrumental variables might be invalid; (3) maximum likelihood method [71]; (4) MR-PRESSO test to identify outliers [72].

To examine the causal relationship between birthweight and breast cancer while considering menarche age and menopausal age as potential confounding factors, we conducted the multivariable inverse-variance weighted method [73, 74]. We also assessed the causal relationship between age at menarche (or/and age at menopause) and breast cancer assuming birthweight was a confounding factor. In order to avoid the impact of horizontal pleiotropy, we relied on a conservative strategy to exclude some candidate instruments that had a *P* value less than 0.05 after Bonferroni’s correction [74–76].

## Results

### Estimated genetic correlation between birthweight and breast cancer

We observed there existed a substantial genetic correlation between fetal-specific birthweight and breast cancer (*r*_*g*_ = 0.123 and *P* = 0.013), in contrast to the negative but non-significant genetic correlation between maternal-specific birthweight and breast cancer (*r*_*g*_ = − 0.068 and *P* = 0.206). This finding is slightly in contrast to results in prior work [43] where neither fetal-specific birthweight nor maternal-specific birthweight was genetically related to breast cancer (*r*_*g*_ = 0.015 and *P* = 0.828 for fetal birthweight; *r*_*g*_ = − 0.072 and *P* = 0.321 for maternal-birthweight); whereas both showed consistent direction for fetal-specific or maternal-specific birthweight. The opposite direction in genetic correlation can be expected as the maternal-specific and fetal-specific SNP effects on birthweight are inversely correlated [43]. In addition, we did not discover significant genetic correlation between birthweight and age at menarche (or age at menopause) (Additional file 1: Table S3).

**Fig. 2:**
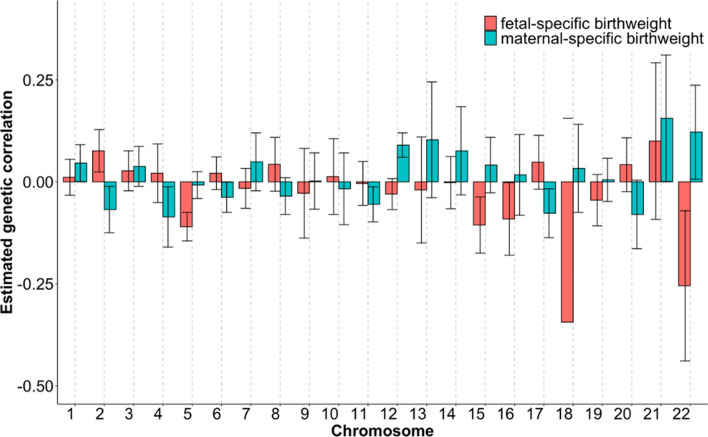
Estimated genetic correlation and 95% confidence intervals for each chromosome between fetal/maternal-specific birthweight and breast cancer

These non-significant genetic correlations do not necessarily imply the absence of shared genetic component between birthweight and breast cancer (or the two ages) as *r*_*g*_ only measures the average genetic correlation of effect sizes for all SNPs across the whole genome, which does not capture detailed patterns for individual shared genetic loci. For example, the mixture of a significantly positive genetic correlation for a local region and a significantly negative genetic correlation for another local region would lead to a non-significant overall genetic correlation, as partly demonstrated by the chromosome-specific genetic correlation in Fig. 2, where both negative and positive relationships were present across the chromosomes. Therefore, we cannot completely rule out the possibility that genes in some local genetic regions would be associated with both birthweight and breast cancer.

### Pleiotropic genes for birthweight and breast cancer

Using MAIUP [59] we identified a large set of commonly associated genes that were shared by birthweight and breast cancer. Specifically, there were 84 pleiotropic genes (false discovery rate [FDR] < 0.05) shared by fetal-specific birthweight and breast cancer (Additional file 1: Table S4), while the number was 49 between maternal-specific birthweight and breast cancer (Additional file 1: Table S5). Among the two sets of pleiotropic associations, there existed 16 common genes including *ANO8*, *BBS1*, *C15orf39*, *CDKAL1*, *DDA1*, *DPP3*, *FAM219B*, *GOLGA6C*, *GTPBP3*, *LOC100652768*, *MPI*, *PCSK7*, *PELI3*, *SCAMP2*, *TAGLN*, and *ZDHHC24*. Several genes were preciously confirmed to have a connection with breast cancer. For example, it was revealed that, together with the molecular subtype, the expression signature of *BBS1* was significantly related to the bone metastasis status of breast cancer and encoded mainly membrane-bound molecules with molecular function of protein binding [77]. A locus, rs9368197, located within *CDKAL1* (intron) was detected to be associated with increased breast cancer risk in European American women [78]. As another example, *TAGLN* was identified as a target of DNA hypermethylation in breast cancer by using microarray expression profiling of AZA- or DMSO-treated breast cancer and non-tumorigenic breast cells [79].

**Fig. 3:**
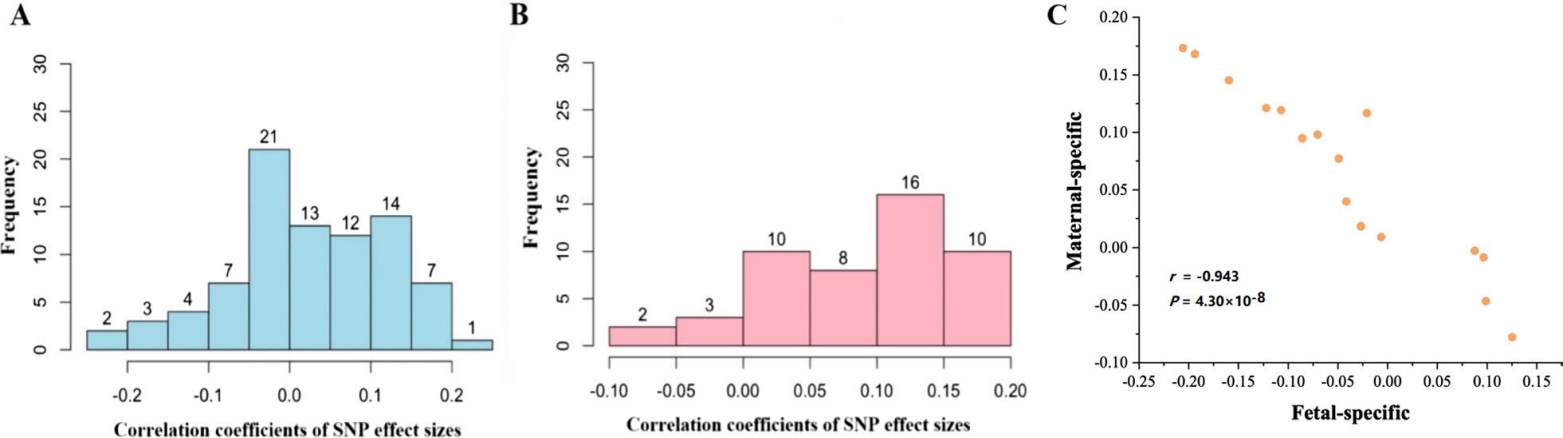
**A** Distribution of correlation coefficients of SNP effect sizes of pleiotropic genes between fetal-specific birthweight and breast cancer. **B** Distribution of correlation coefficients of SNP effect sizes of pleiotropic genes between maternal-specific birthweight and breast cancer. **C** Relationship between correlation coefficients of SNP effect sizes for the 16 genes shared by fetal-specific birthweight with breast cancer and these of SNP effect sizes for the 16 genes shared by maternal-specific birthweight with breast cancer

To evaluate the similarity of genetic influence of these pleiotropic genes, for every shared gene we further calculated the Pearson’s correlation coefficient of effect sizes between birthweight and breast cancer with local SNPs belonging to that gene. We found that most of these pleiotropic genes (~ 68.4%)—47 out of 84 for fetal-specific birthweight and 44 out of 49 for maternal-specific birthweight—displayed a positive correlation in effect direction, meaning that they generally exerted consistent genetic impact on birthweight and breast cancer (Fig. 3A, B). These genes with consistent effects are believed to contribute to the observed positive relationship between birthweight and breast cancer. The remaining pleiotropic genes demonstrated a negative correlation in SNP effect sizes between the two traits, indicating that these genes exhibit functionally different influence on birthweight and breast cancer. Note that, this phenomenon of antagonistic effects of shared genetic loci is also widely observed for other genetically correlated traits such as psychiatric disorders [80, 81] and immune-mediated diseases [82–84].

We further compared the correlation coefficients for the 16 genes shared by fetal/maternal-specific birthweight with breast cancer. It is very interesting that the two sets of correlation coefficients were completely opposite, with a highly negative correlation between themselves (Fig. 3C), indicating that these pleiotropic genes showed considerably distinct genetic impact on birthweight and breast cancer. For example, the genes *FAM219B* and *TAGLN* presented a negative correlation of SNP effect sizes between fetal-specific birthweight and breast cancer (*r* = − 0.122 and 0.194, respectively), but displayed a positive correlation between maternal-specific birthweight and breast cancer (*r* = 0.121 and 0.168, respectively).

### Estimated causal effect with MR analysis

We conducted a set of MR analyses to assess the causal association between birthweight, breast cancer, age at menarche, and age at menopause. It is worth mentioning that we had chosen two different sets of instruments for birthweight: one set of SNPs with fetal-specific effect on birthweight and another set of SNPs with maternal-specific effect on birthweight (Additional file 1: Tables S1, S2), which offered us an effective manner to untangle the origin of the observed relationship between birthweight and breast cancer. First, we carried out the univariate inverse-variance weighted (IVW) analysis to evaluate the impact birthweight on breast cancer, but failed to find evidence of causal relationship between birthweight and breast cancer (*P* = 0.806 for fetal-specific birthweight, *P* = 0.244 for the maternal-specific birthweight). We conducted an online simulation with the same sample size and proportion of breast cancer cases used here [85]. As a result, we had a statistical power of 72% when the odds ratio was assumed to be 0.90 for fetal-specific birthweight and breast cancer or 86% for maternal-specific birthweight and breast cancer at the significance level of 0.05 (Additional file 1: Figure S1). This simulation finding indicated that our MR analysis had moderate or high power in discovering a significant association. Therefore, it to a great extent ruled out the likelihood that the null causal association between fetal/maternal-specific birthweight and breast cancer observed above was due to low power. Then, we performed the univariate IVW analysis to assess the association between birthweight and age at menarche, and observed that fetal-specific birthweight was positively correlated to age at menarche (*β* = 0.089 and *P* = 0.012), indicating higher birthweight can delay age at menarche in a fetal way; but we did not detect a substantial association between maternal-specific birthweight and age at menarche (*P* = 0.907).

These results were also replicated in various MR sensitivity analyses (Additional file 1: Table S7). For instance, compared to the IVW method, the weighted median method and the maximum likelihood method produced similar causal estimates. In addition, based on MR-PRESSO, we did not observed no horizontal pleiotropy in the association analyses of fetal-specific birthweight and age at menarche (*P*_outlier_ = 0.576), fetal-birthweight and age at menopause (*P*_outlier_ = 0.122), as well as age at menarche and age at menopause (*P*_outlier_ = 0.773). For these cases with horizontal pleiotropy, we removed outlier instruments and still obtained similar effect estimates as before (Additional file 1: Table S8).

Next, we conducted the multivariate IVW analysis to assess the relationship between birthweight and age at menopause while controlling the influence of age at menarche. We discovered that there was no substantial causal association between birthweight and age at menopause (*P* = 0.927 for the fetal-specific birthweight and *P* = 0.590 for the maternal-specific birthweight); however, we found that age at menarche was a significant confounder with a positive effect on age at menopause for fetal-specific birthweight (*β* = 0.111 and *P* = 0.035), while this association was not significant for maternal-specific birthweight (*β* = 0.123 and *P* = 0.062). This suggests that fetal-specific birthweight might play a more important role than maternal-specific birthweight in the relationship between age at menarche and age at menopause. Note that, age at menarche can influence age at menopause but not vice versa. We also examined the relationship between age at menarche and breast cancer while adjusting for birthweight but did not observe obviously causal association between them (*P* = 0.525 when controlling for fetal-specific birthweight, and *P* = 0.368 when controlling for maternal-specific birthweight).

**Fig. 4:**
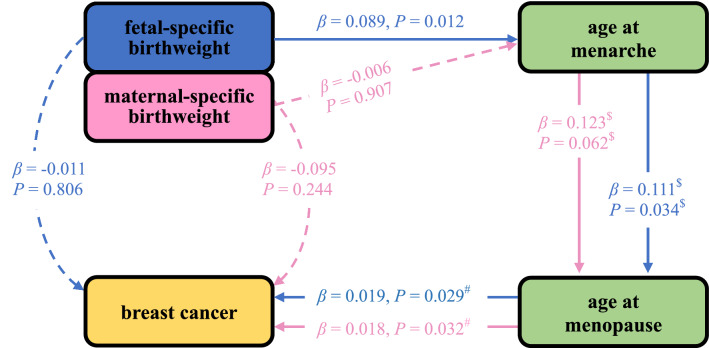
Estimated effect sizes and corresponding *P* values in the MR analysis. $ indicates the estimated effect sizes were obtained while adjusting for fetal-specific or maternal-specific birthweight; # denotes the estimated effect sizes were obtained while controlling for fetal/maternal-specific birthweight and age at menarche. Note here that the color of arrow and number is consistent with that for fetal-specific or maternal-specific birthweight. Dot arrow stands for the absence of association, while solid arrow stands for the presence of association. To be concise and easy to understand, besides all significant associations, only a few of important null associations are shown

Finally, we evaluated the relationship between age at menopause and breast cancer while controlling for birthweight and age at menarche. We discovered that there only existed a significant positive correlation between age at menopause and breast cancer (*P* = 0.029 for when adjusting for fetal-specific birthweight and age at menarche, *P* = 0.032 for when adjusting for maternal-specific birthweight and age at menarche). Again, according to the naïve principle of mediation analysis, we can conclude that age at menarche and age at menopause mediated the impact of birthweight on adult breast cancer, among which the fetal role appeared much more evident. The associations identified by distinct MR analyses are demonstrated in Fig. 4, with the detailed results further shown in Additional file 1: Table S6. Note that, although we conducted several multivariate MR analyses in the mediation analysis above, we did not consider the issue of multiple testing when assessing the association between the exposure (i.e., fetal/maternal birthweight) and the mediator (i.e., age at menarche or age at menopause) and the association between the mediator and the outcome (i.e., breast cancer). The reason was that the mediator was assumed to exert a mediating impact if and only if both the two associations needed to be significant in terms of the essential rationale of mediation analysis [46, 86, 87].

## Discussion

In the present study we have investigated the genetic correlation and causal association between birthweight and breast cancer. To our knowledge, the present work is among the first endeavor to study the relationship between the two traits by leveraging novel statistical methods with large-scale summary-level genetic data. As a result, we offered implicit answers for some key questions regarding such relationship between them. First, to understand whether the observed relationship is due to common genetic background, we employed LDSC [44] and identified a positively significant genetic correlation between fetal-specific birthweight and breast cancer as well as a negative but insignificant genetic correlation between maternal-specific birthweight and breast cancer. Moreover, using MAIUP [59] we showed that there were extensively common genetic loci underlying the two traits. Second, to examine whether the observed relationship represents a linear causality between birthweight and breast cancer, we carried out the MR analysis but did not identify a linear causal association, which is in agreement with the null finding obtained from another MR study [39]. Third, to determine whether some growth traits and life processes may mediate the long-term impact of birthweight on breast cancer, we depended on the principle of mediation analysis [46, 86, 88–90] and demonstrated that fetal-specific birthweight can indirectly influence breast cancer risk in adulthood via the path of age of menarche or age at menopause.

Unlike prior relevant studies [39], one of the remarkable strengths of our work is that we resolved the relative contributions of fetal and maternal genotypes on birthweight and employed fetal/maternal-specific effects of birthweight in our genetic overlap analysis as well as in our MR analysis, which provides us an unprecedented opportunity to untangle the origin of the relationship between birthweight and breast cancer [43, 66, 67]. For example, we discovered that fetal-specific birthweight was genetically correlated to breast cancer in a positive direction, while maternal-specific birthweight showed a negative genetic correlation to breast cancer. In addition, as demonstrated, the pleiotropic genes shared between fetal-specific birthweight and breast cancer was not completely overlapped with those shared between maternal-specific birthweight and breast cancer, implying the diverse contribution of fetal-specific birthweight and maternal-specific birthweight to the observed relationship. Furthermore, we found that fetal-specific birthweight, rather than maternal-specific birthweight, was causally associated with age of menarche which further would affect age at menopause and breast cancer, indicating that, together the identified positive genetic correlation mentioned above, fetal-specific birthweight might exert a more pronounced influence on the development of breast cancer in later life compared to maternal-specific birthweight. Together, these findings suggest that the growth environment in childhood might be very important for the development of breast cancer in adulthood. However, there is insufficient evidence of maternal-specific birthweight effect on offspring's breast cancer, implying that the maternal intrauterine environment does not seem to be the major determinant of the risk of breast cancer.

Some limitations of the current work should be mentioned. First, like other MR studies, we assumed a linear effect association between birthweight and breast cancer but cannot completely rule out the likelihood of nonlinear association between birthweight and breast cancer as suggested in prior studies [15, 29]. Second, no data on the duration and severity of breast cancer can be available for us; therefore, we cannot assess the dose–response association between birthweight and breast cancer, which is an important aspect of causal inference. Third, due to unavailability of relevant data, we cannot further assess the impact of birthweight on distinct subtypes of breast cancer. Because of the same reason, we also cannot evaluate the association between birthweight and breast cancer stratified by the menopausal status [15], which may indicate various influence of birthweight on breast cancer [22, 23, 91]. Fourth, because the traditional mediation test methods such as the Sobel test and the joint significance test [46], in our analysis we did employ any formal approaches but only applied the naïve principle that the presence of both the exposure-mediator effect and mediator-outcome effect indicates the existence of mediation effect. Therefore, powerful mediation test methods would be warranted for a single or only a few mediators under the summary-level framework, which is our ongoing work. Fifth, we here only two mediators (e.g., age of menarche and age at menopause) were considered; a more comprehensive evaluation of growth traits and life processes are projected to discover other causal paths from birthweight to breast cancer.

Overall, this study reveals that shared genetic foundation and causal mediation commonly drive the connection between the two traits, and that fetal/maternal-specific birthweight plays substantially distinct roles in such relationship. However, our work offers little supportive evidence for the fetal origins hypothesis of breast cancer originating in utero.

## Supplementary Information

**Additional file 1.** Supplementary files.

## Data Availability

All data generated or analyzed during this study are included in this article and its additional information files.

## Abbreviations

LDSC: Linkage disequilibrium score regression
GWAS: Genome-wide association study
MAIUP: Mixture Adjusted Intersect-Union Pleiotropy test
MR: Mendelian randomization
MAGMA: Multi-marker Analysis of GenoMic Annotation
IVW: Inverse variance weighted method
SNP: Single nucleotide polymorphism
