# Supplementary material for: Exploring the association between birthweight and breast cancer using summary statistics from a perspective of genetic correlation, mediation, and causality
Source: J Transl Med. 2022 May 14;20:227. doi: 10.1186/s12967-022-03435-2 (PMC9107660; doi:10.1186/s12967-022-03435-2)
Supplement: Supplementary file 1 — Additional file 1. Supplementary files. [file 12967_2022_3435_MOESM1_ESM.docx]

# Additional File

## Table S1. Instrumental variables for fetal-specific birthweight

| SNP | chr | position | effect  allele | non-effect  allele | *β* | se | *P* |
| --- | --- | --- | --- | --- | --- | --- | --- |
| rs12401656 | 1 | 43456767 | G | A | 0.029 | 0.006 | 1.60×10^-6^ |
| rs80278614 | 1 | 119412317 | A | G | 0.052 | 0.009 | 4.00×10^-8^ |
| rs10913200 | 1 | 176521655 | G | A | 0.038 | 0.013 | 2.60×10^-3^ |
| rs61830764 | 1 | 212289976 | A | G | 0.018 | 0.004 | 3.00×10^-5^ |
| rs10495563 | 2 | 9662210 | A | G | 0.016 | 0.004 | 2.80×10^-4^ |
| rs11893688 | 2 | 9695282 | T | C | 0.015 | 0.004 | 4.50×10^-4^ |
| rs2551347 | 2 | 23912401 | T | C | 0.029 | 0.005 | 2.20×10^-9^ |
| rs754868 | 2 | 43185532 | G | A | 0.019 | 0.004 | 4.70×10^-6^ |
| rs17034876 | 2 | 46484310 | T | C | 0.039 | 0.005 | 5.50×10^-17^ |
| rs4953353 | 2 | 46567276 | G | T | 0.019 | 0.004 | 6.50×10^-6^ |
| rs56188432 | 2 | 158406865 | G | A | 0.250 | 0.049 | 2.60×10^-7^ |
| rs560887 | 2 | 169763148 | C | T | -0.025 | 0.004 | 2.80×10^-8^ |
| rs10181515 | 2 | 227019461 | T | C | 0.021 | 0.005 | 1.30×10^-5^ |
| rs9855896 | 3 | 14287150 | G | A | -0.014 | 0.005 | 6.10×10^-3^ |
| rs2168443 | 3 | 46947087 | T | A | 0.010 | 0.004 | 0.015 |
| rs11708067 | 3 | 123065778 | G | A | 0.056 | 0.005 | 6.30×10^-32^ |
| rs10935733 | 3 | 148622968 | T | C | 0.021 | 0.004 | 5.00×10^-7^ |
| rs4679760 | 3 | 155855418 | G | C | -0.009 | 0.004 | 0.033 |
| rs1482852 | 3 | 156798294 | A | G | 0.054 | 0.004 | 7.60×10^-39^ |
| rs11711420 | 3 | 183349010 | T | G | 0.022 | 0.005 | 2.70×10^-6^ |
| rs4144829 | 4 | 17903654 | C | T | 0.032 | 0.005 | 1.10×10^-11^ |
| rs2174633 | 4 | 17917781 | A | C | 0.031 | 0.005 | 3.60×10^-11^ |
| rs116807401 | 4 | 135121721 | C | T | 0.088 | 0.016 | 7.00×10^-8^ |
| rs6845999 | 4 | 145565826 | T | C | 0.017 | 0.004 | 2.80×10^-5^ |
| rs2131354 | 4 | 145599908 | A | G | 0.016 | 0.004 | 1.90×10^-4^ |
| rs351930 | 5 | 52003397 | T | A | 0.020 | 0.005 | 8.10×10^-5^ |
| rs35261542 | 6 | 20675792 | C | A | 0.049 | 0.005 | 3.20×10^-26^ |
| rs9267812 | 6 | 32128394 | T | C | 0.015 | 0.006 | 0.012 |
| rs1547669 | 6 | 33775641 | G | A | 0.018 | 0.004 | 9.30×10^-6^ |
| rs75104038 | 6 | 34190104 | A | G | 0.024 | 0.009 | 6.00×10^-3^ |
| rs75034466 | 6 | 34199815 | T | C | 0.020 | 0.010 | 0.040 |
| rs6925689 | 6 | 126865884 | T | C | 0.018 | 0.004 | 2.10×10^-5^ |
| rs6930558 | 6 | 141878920 | T | G | 0.022 | 0.005 | 3.30×10^-6^ |
| rs10872678 | 6 | 152039964 | T | C | 0.028 | 0.005 | 8.20×10^-10^ |
| rs7772579 | 6 | 152042502 | A | C | 0.027 | 0.005 | 5.80×10^-9^ |
| rs34776209 | 7 | 23513093 | C | T | 0.015 | 0.005 | 1.30×10^-3^ |
| rs138715366 | 7 | 44246271 | C | T | 0.235 | 0.022 | 1.40×10^-25^ |
| rs10265133 | 7 | 45895604 | G | T | -0.020 | 0.006 | 9.70×10^-4^ |
| rs10265057 | 7 | 47275737 | G | A | 0.036 | 0.007 | 4.60×10^-7^ |
| rs2237467 | 7 | 50733316 | A | G | 0.011 | 0.005 | 0.022 |
| rs112139215 | 7 | 73034559 | A | C | 0.056 | 0.008 | 1.20×10^-11^ |
| rs2282978 | 7 | 92264410 | C | T | 0.021 | 0.004 | 1.60×10^-6^ |
| rs13231367 | 7 | 127509070 | G | A | 0.009 | 0.005 | 0.047 |
| rs6467157 | 7 | 127660763 | T | C | 0.014 | 0.005 | 3.00×10^-3^ |
| rs732563 | 8 | 23345526 | C | T | 0.019 | 0.004 | 6.10×10^-6^ |
| rs34036147 | 8 | 38366249 | T | C | 0.019 | 0.004 | 1.60×10^-5^ |
| rs13266210 | 8 | 41533514 | A | G | 0.030 | 0.005 | 3.10×10^-9^ |
| rs72656010 | 8 | 57122215 | T | C | 0.026 | 0.006 | 1.60×10^-5^ |
| rs6995390 | 8 | 77611012 | T | A | -0.014 | 0.006 | 0.010 |
| rs7819593 | 8 | 106115172 | C | T | 0.023 | 0.005 | 2.10×10^-6^ |
| rs9657468 | 8 | 142362391 | G | T | 0.018 | 0.004 | 3.60×10^-5^ |
| rs28457693 | 9 | 98217348 | G | A | 0.040 | 0.007 | 1.70×10^-9^ |
| rs1411424 | 9 | 113892963 | A | G | 0.012 | 0.004 | 3.90×10^-3^ |
| rs2418135 | 9 | 113901309 | A | G | 0.012 | 0.004 | 2.90×10^-3^ |
| rs72760655 | 9 | 116916214 | C | A | -0.009 | 0.004 | 0.044 |
| rs1323438 | 9 | 119115531 | C | T | 0.020 | 0.005 | 1.30×10^-5^ |
| rs3933326 | 9 | 123633948 | G | A | 0.023 | 0.004 | 2.20×10^-7^ |
| rs28505901 | 9 | 139241030 | A | G | 0.024 | 0.005 | 4.20×10^-7^ |
| rs5030938 | 10 | 70975916 | T | C | 0.019 | 0.004 | 1.60×10^-5^ |
| rs9645500 | 10 | 70986723 | G | T | 0.019 | 0.004 | 1.00×10^-5^ |
| rs1112718 | 10 | 94479107 | G | A | 0.036 | 0.004 | 1.50×10^-17^ |
| rs10509669 | 10 | 95969913 | A | T | -0.020 | 0.005 | 2.60×10^-5^ |
| rs562974282 | 10 | 104201070 | G | T | -0.126 | 0.054 | 0.019 |
| rs7076938 | 10 | 115789375 | T | C | 0.029 | 0.005 | 2.90×10^-10^ |
| rs71486610 | 10 | 124134803 | C | G | 0.016 | 0.004 | 1.70×10^-4^ |
| rs11042596 | 11 | 2118860 | T | G | 0.027 | 0.004 | 1.60×10^-9^ |
| rs234864 | 11 | 2857297 | A | G | 0.017 | 0.004 | 4.90×10^-5^ |
| rs2168101 | 11 | 8255408 | C | A | -0.015 | 0.005 | 1.00×10^-3^ |
| rs4444073 | 11 | 10331664 | A | C | 0.023 | 0.004 | 2.20×10^-8^ |
| rs667515 | 11 | 69449076 | G | C | 0.013 | 0.004 | 1.50×10^-3^ |
| rs76895963 | 12 | 4384844 | G | T | 0.051 | 0.016 | 1.30×10^-3^ |
| rs11055030 | 12 | 12878349 | G | C | 0.022 | 0.005 | 1.00×10^-6^ |
| rs8756 | 12 | 66359752 | C | A | 0.037 | 0.004 | 1.70×10^19^ |
| rs7968682 | 12 | 66371880 | G | T | 0.037 | 0.004 | 4.90×10^-20^ |
| rs1480470 | 12 | 66412130 | G | A | 0.028 | 0.004 | 1.10×10^-10^ |
| rs2647873 | 12 | 103081192 | A | G | 0.009 | 0.004 | 0.033 |
| rs34217484 | 13 | 48854550 | A | T | 0.012 | 0.005 | 0.011 |
| rs72681869 | 14 | 50655357 | C | G | 0.108 | 0.021 | 2.70×10^-7^ |
| rs6575803 | 14 | 101257755 | C | T | 0.034 | 0.007 | 9.90×10^-7^ |
| rs75844534 | 15 | 38667117 | A | C | 0.036 | 0.006 | 1.50×10^-8^ |
| rs339969 | 15 | 60883281 | A | C | 0.011 | 0.004 | 0.012 |
| rs3784789 | 15 | 75082552 | G | C | -0.018 | 0.004 | 2.40×10^-5^ |
| rs4932373 | 15 | 91429287 | A | C | 0.010 | 0.004 | 0.021 |
| rs7402983 | 15 | 99193276 | A | C | 0.027 | 0.004 | 4.60×10^-10^ |
| rs40434 | 16 | 55699525 | G | A | 0.017 | 0.004 | 4.80×10^-5^ |
| rs222857 | 17 | 7164563 | T | C | 0.026 | 0.004 | 5.80×10^-10^ |
| rs4511593 | 17 | 7455536 | T | C | 0.019 | 0.004 | 7.40×10^-6^ |
| rs9909342 | 17 | 25652275 | A | G | 0.019 | 0.004 | 6.70×10^-6^ |
| rs11867479 | 17 | 68090207 | T | C | 0.018 | 0.004 | 2.20×10^-5^ |
| rs10221267 | 17 | 68464662 | T | C | 0.018 | 0.004 | 1.90×10^-5^ |
| rs73354194 | 17 | 79905947 | C | T | 0.060 | 0.014 | 1.70×10^-5^ |
| rs8106042 | 19 | 7161849 | G | C | 0.023 | 0.005 | 6.60×10^-7^ |
| rs41355649 | 19 | 33790556 | G | A | 0.042 | 0.008 | 4.50×10^-7^ |
| rs1129156 | 19 | 40719076 | T | C | 0.022 | 0.005 | 1.90×10^-6^ |
| rs147957154 | 19 | 43431040 | T | C | 0.026 | 0.006 | 2.40×10^-5^ |
| rs1203876 | 20 | 22540915 | C | A | 0.055 | 0.010 | 1.30×10^-8^ |
| rs11698914 | 20 | 31327144 | C | G | 0.029 | 0.005 | 2.80×10^-9^ |
| rs1012167 | 20 | 39159119 | C | T | 0.024 | 0.004 | 1.90×10^-8^ |
| rs753381 | 20 | 39797465 | T | C | 0.018 | 0.004 | 9.10×10^-6^ |
| rs6026449 | 20 | 57272617 | C | T | 0.018 | 0.004 | 3.20×10^-5^ |
| rs73143584 | 20 | 62445702 | A | G | 0.031 | 0.007 | 3.30×10^-6^ |
| rs134594 | 22 | 29468456 | C | T | 0.022 | 0.004 | 6.00×10^-7^ |
| rs41311445 | 22 | 42070374 | A | C | 0.034 | 0.007 | 1.30×10^-6^ |
| rs7285579 | 22 | 46441980 | C | T | 0.018 | 0.005 | 1.10×10^-4^ |

## Table S2. Instrumental variables for maternal-specific birthweight

| SNP | chr | position | effect  allele | non-effect  allele | *β* | se | *P* |
| --- | --- | --- | --- | --- | --- | --- | --- |
| rs17367504 | 1 | 11862778 | G | A | 0.032 | 0.006 | 2.50×10^-7^ |
| rs10913200 | 1 | 176521655 | G | A | 0.029 | 0.014 | 0.041 |
| rs10495563 | 2 | 9662210 | A | G | 0.013 | 0.005 | 7.70×10^-3^ |
| rs11893688 | 2 | 9695282 | T | C | 0.013 | 0.005 | 6.30×10^-3^ |
| rs4952673 | 2 | 43423870 | A | G | 0.025 | 0.005 | 1.20×10^-7^ |
| rs17034876 | 2 | 46484310 | T | C | 0.011 | 0.005 | 0.030 |
| rs560887 | 2 | 169763148 | C | T | 0.038 | 0.005 | 5.40×10^-14^ |
| rs9855896 | 3 | 14287150 | G | A | 0.033 | 0.006 | 2.60×10^-9^ |
| rs2168443 | 3 | 46947087 | T | A | 0.011 | 0.005 | 0.019 |
| rs11708067 | 3 | 123065778 | G | A | -0.029 | 0.005 | 3.80×10^-8^ |
| rs9851257 | 3 | 123125711 | T | A | 0.027 | 0.005 | 1.10×10^-6^ |
| rs6440006 | 3 | 141142691 | A | G | 0.020 | 0.005 | 1.30×10^-5^ |
| rs4679760 | 3 | 155855418 | G | C | 0.038 | 0.005 | 6.50×10^-15^ |
| rs2174633 | 4 | 17917781 | A | C | 0.011 | 0.005 | 0.039 |
| rs2189234 | 4 | 106075498 | G | T | 0.026 | 0.005 | 7.30×10^-8^ |
| rs6845999 | 4 | 145565826 | T | C | 0.017 | 0.005 | 3.70×10^-4^ |
| rs2131354 | 4 | 145599908 | A | G | 0.019 | 0.005 | 7.50×10^-5^ |
| rs4579095 | 4 | 174726635 | G | A | 0.023 | 0.005 | 1.50×10^-6^ |
| rs2946179 | 5 | 157886627 | C | T | 0.045 | 0.005 | 3.70×10^-17^ |
| rs34471628 | 5 | 172196752 | A | G | 0.067 | 0.012 | 5.20×10^-8^ |
| rs9379084 | 6 | 7231843 | G | A | 0.040 | 0.007 | 4.70×10^-8^ |
| rs35261542 | 6 | 20675792 | C | A | -0.019 | 0.005 | 6.10×10^-4^ |
| rs6911024 | 6 | 31368451 | T | C | 0.040 | 0.008 | 2.20×10^-7^ |
| rs9267812 | 6 | 32128394 | T | C | 0.014 | 0.007 | 0.048 |
| rs75104038 | 6 | 34190104 | A | G | 0.041 | 0.010 | 3.60×10^-5^ |
| rs75034466 | 6 | 34199815 | T | C | 0.051 | 0.011 | 5.60×10^-6^ |
| rs34776209 | 7 | 23513093 | C | T | 0.014 | 0.006 | 9.60×10^-3^ |
| rs2971669 | 7 | 44231778 | T | C | 0.028 | 0.006 | 4.90×10^-7^ |
| rs10265133 | 7 | 45895604 | G | T | 0.034 | 0.007 | 8.90×10^-7^ |
| rs2237467 | 7 | 50733316 | A | G | 0.012 | 0.006 | 0.029 |
| rs45446698 | 7 | 99332948 | G | T | 0.077 | 0.012 | 1.10×10^-10^ |
| rs13231367 | 7 | 127509070 | G | A | 0.018 | 0.005 | 4.80×10^-4^ |
| rs6467157 | 7 | 127660763 | T | C | 0.014 | 0.005 | 4.90×10^-3^ |
| rs3918226 | 7 | 150690176 | C | T | 0.040 | 0.009 | 7.40×10^-6^ |
| rs11778247 | 8 | 23403378 | G | A | 0.026 | 0.006 | 6.20×10^-5^ |
| rs6995390 | 8 | 77611012 | T | A | 0.039 | 0.006 | 3.90×10^-10^ |
| rs1411424 | 9 | 113892963 | A | G | 0.016 | 0.005 | 6.10×10^-4^ |
| rs2418135 | 9 | 113901309 | A | G | 0.015 | 0.005 | 9.10×10^-4^ |
| rs72760655 | 9 | 116916214 | C | A | 0.031 | 0.005 | 7.60×10^-10^ |
| rs5030938 | 10 | 70975916 | T | C | 0.010 | 0.005 | 0.045 |
| rs9645500 | 10 | 70986723 | G | T | 0.010 | 0.005 | 0.042 |
| rs1112718 | 10 | 94479107 | G | A | -0.018 | 0.005 | 1.70×10^-4^ |
| rs10509669 | 10 | 95969913 | A | T | 0.039 | 0.005 | 3.60×10^-13^ |
| rs3740360 | 10 | 96025491 | C | A | 0.044 | 0.007 | 1.60×10^-9^ |
| rs562974282 | 10 | 104201070 | G | T | 0.269 | 0.055 | 9.60×10^-7^ |
| rs71486610 | 10 | 124134803 | C | G | 0.011 | 0.005 | 0.024 |
| rs2168101 | 11 | 8255408 | C | A | 0.039 | 0.005 | 5.90×10^-14^ |
| rs10734564 | 11 | 48160429 | A | G | 0.033 | 0.006 | 1.20×10^-7^ |
| rs667515 | 11 | 69449076 | G | C | 0.011 | 0.005 | 0.026 |
| rs10830963 | 11 | 92708710 | G | C | 0.046 | 0.005 | 4.60×10^-19^ |
| rs10895278 | 11 | 102095335 | C | T | 0.025 | 0.005 | 3.30×10^-7^ |
| rs76895963 | 12 | 4384844 | G | T | 0.040 | 0.018 | 0.030 |
| rs11051061 | 12 | 30914668 | A | G | 0.026 | 0.005 | 1.10×10^-6^ |
| rs180438 | 12 | 47187260 | G | A | 0.039 | 0.006 | 4.10×10^-11^ |
| rs1533688 | 12 | 102772745 | C | T | 0.025 | 0.006 | 1.60×10^-5^ |
| rs2647873 | 12 | 103081192 | A | G | 0.017 | 0.005 | 3.30×10^-4^ |
| rs17033114 | 12 | 103123339 | T | C | 0.053 | 0.010 | 6.20×10^-8^ |
| rs3184504 | 12 | 111884608 | C | T | 0.034 | 0.005 | 1.80×10^-13^ |
| rs34217484 | 13 | 48854550 | A | T | 0.013 | 0.005 | 0.013 |
| rs75844534 | 15 | 38667117 | A | C | -0.021 | 0.007 | 2.90×10^-3^ |
| rs2928148 | 15 | 41401550 | A | G | 0.020 | 0.005 | 1.60×10^-5^ |
| rs339969 | 15 | 60883281 | A | C | 0.010 | 0.005 | 0.036 |
| rs3784789 | 15 | 75082552 | G | C | 0.030 | 0.005 | 1.20×10^-9^ |
| rs12909648 | 15 | 86224570 | G | A | 0.027 | 0.005 | 3.40×10^-9^ |
| rs12443252 | 15 | 91064690 | T | C | 0.023 | 0.005 | 1.70×10^-6^ |
| rs4932373 | 15 | 91429287 | A | C | 0.019 | 0.005 | 1.70×10^-4^ |
| rs11641308 | 16 | 75312023 | T | C | 0.023 | 0.005 | 7.90×10^-6^ |
| rs2967676 | 19 | 8789666 | A | C | 0.048 | 0.006 | 4.40×10^-14^ |
| rs12461110 | 19 | 56320663 | A | G | 0.022 | 0.005 | 3.40×10^-6^ |
| rs304001 | 19 | 56423668 | G | A | 0.023 | 0.005 | 1.90×10^-6^ |
| rs1203876 | 20 | 22540915 | C | A | -0.040 | 0.011 | 1.60×10^-4^ |
| rs181451002 | 20 | 32466219 | G | A | 0.059 | 0.016 | 3.10×10^-4^ |

## Table S3. Estimated genetic correlation between birthweight and breast cancer

| exposure | outcome | *r_g_* | se | z | *P* |
| --- | --- | --- | --- | --- | --- |
| *X*_1_ | *M*_1_ | -0.004 | 0.010 | -0.387 | 0.699 |
| *X*_2_ | *M*_1_ | -0.003 | 0.010 | -0.306 | 0.760 |
| *X*_1_ | *M*_2_ | 0.088 | 0.059 | 1.476 | 0.140 |
| *X*_2_ | *M*_2_ | -0.073 | 0.055 | -1.324 | 0.186 |
| *X*_1_ | *Y* | 0.123 | 0.049 | 2.485 | 0.013 |
| *X*_2_ | *Y* | -0.068 | 0.053 | -1.266 | 0.206 |

Note**:** *X*_1_: fetal-specific birthweight; *X*_2_: maternal-specific birthweight; *M*_1_: age at menarche; *M*_2_: age at menopause; *Y*: breast cancer.

## Table S4. Pleiotropic genes for fetal-specific birthweight and breast cancer detected by MAIUP

| gene | chr | start ~ stop | *P* (birthweight) | *P* (breast cancer) | FDR | Pearson |
| --- | --- | --- | --- | --- | --- | --- |
| *ABHD8* | 19 | 17302939~17514282 | 5.11×10^-4^ | 5.89×10^-5^ | 1.62×10^-3^ | -0.023 |
| *ACADVL* | 17 | 7020443~7228586 | 1.59×10^-10^ | 9.52×10^-5^ | 2.11×10^-5^ | -0.024 |
| *ACAP1* | 17 | 7139847~7354793 | 1.18×10^-7^ | 8.18×10^-7^ | 5.12×10^-3^ | 0.122 |
| *ACTN3* | 11 | 66213865~66430800 | 1.77×10^-4^ | 1.60×10^-5^ | 7.85×10^-3^ | 0.123 |
| *ADM* | 11 | 10226641~10428923 | 1.06×10^-4^ | 2.55×10^-4^ | 2.52×10^-3^ | 0.012 |
| *ANO8* | 19 | 17334031~17545638 | 4.75×10^-4^ | 5.61×10^-5^ | 9.23×10^-3^ | -0.086 |
| *ARHGEF2* | 1 | 155816629~156048336 | 4.80×10^-4^ | 1.46×10^-6^ | 2.52×10^-3^ | 0.065 |
| *BBS1* | 11 | 66178118~66401084 | 8.67×10^-5^ | 1.22×10^-5^ | 5.43×10^-3^ | 0.099 |
| *BCAP29* | 7 | 107120421~107363762 | 3.88×10^-4^ | 4.46×10^-4^ | 5.70×10^-3^ | 0.020 |
| *C15orf39* | 15 | 75394220~75604510 | 9.07×10^-5^ | 5.10×10^-5^ | 9.33×10^-5^ | -0.027 |
| *CCDC170* | 6 | 151715174~152042328 | 9.81×10^-5^ | 7.06×10^-32^ | 1.72×10^-4^ | 0.034 |
| *CCDC85B* | 11 | 65557874~65759106 | 5.30×10^-4^ | 8.25×10^-11^ | 4.98×10^-3^ | 0.021 |
| *CCDC87* | 11 | 66257639~66460554 | 1.90×10^-4^ | 5.76×10^-5^ | 8.35×10^-3^ | 0.164 |
| *CCS* | 11 | 66260689~66473490 | 2.06×10^-4^ | 5.57×10^-5^ | 5.15×10^-3^ | 0.163 |
| *CDKAL1* | 6 | 20434687~21332634 | 9.48×10^-21^ | 1.07×10^-8^ | 6.80×10^-3^ | -0.006 |
| *CLDN7* | 17 | 7063221~7266512 | 3.06×10^-10^ | 4.37×10^-6^ | 1.67×10^-3^ | -0.042 |
| *CTDNEP1* | 17 | 7046905~7255259 | 2.80×10^-10^ | 1.71×10^-5^ | 2.17×10^-5^ | -0.041 |
| *CTSF* | 11 | 66230934~66436047 | 1.69×10^-4^ | 6.11×10^-5^ | 1.04×10^-3^ | 0.122 |
| *CTSW* | 11 | 65547283~65751212 | 6.70×10^-4^ | 2.86×10^-11^ | 9.33×10^-5^ | 0.008 |
| *DDA1* | 19 | 17320336~17534106 | 5.10×10^-4^ | 1.66×10^-4^ | 2.84×10^-3^ | -0.042 |
| *DLG4* | 17 | 6993209~7223369 | 1.32×10^-8^ | 3.32×10^-4^ | 9.38×10^-4^ | -0.020 |
| *DPP3* | 11 | 66147483~66377130 | 1.74×10^-8^ | 8.15×10^-6^ | 4.81×10^-3^ | 0.097 |
| *DUS4L* | 7 | 107104401~107318968 | 2.67×10^-4^ | 3.40×10^-4^ | 8.02×10^-4^ | 0.013 |
| *DVL2* | 17 | 7028660~7237863 | 8.40×10^-11^ | 1.16×10^-4^ | 4.66×10^-4^ | -0.038 |
| *E2F3* | 6 | 20302136~20593945 | 6.20×10^-10^ | 4.57×10^-6^ | 2.11×10^-5^ | 0.034 |
| *EIF5A* | 17 | 7110317~7315782 | 7.86×10^-8^ | 3.10×10^-5^ | 1.80×10^-4^ | 0.106 |
| *ELP5* | 17 | 7055371~7263259 | 1.52×10^-10^ | 4.74×10^-6^ | 3.81×10^-3^ | -0.037 |
| *ESR1* | 6 | 151911630~152524408 | 4.41×10^-6^ | 6.31×10^-41^ | 7.33×10^-3^ | 0.042 |
| *FAM219B* | 15 | 75092327~75299462 | 6.36×10^-4^ | 7.47×10^-5^ | 9.23×10^-3^ | -0.122 |
| *FOSL1* | 11 | 65559691~65767997 | 6.62×10^-4^ | 2.17×10^-10^ | 1.93×10^-3^ | 0.030 |
| *FRYL* | 4 | 48399379~48882316 | 8.22×10^-5^ | 4.57×10^-4^ | 6.99×10^-3^ | -0.026 |
| *GABARAP* | 17 | 7043737~7245753 | 6.08×10^-11^ | 5.56×10^-5^ | 3.94×10^-3^ | -0.048 |
| *GOLGA6C* | 15 | 75450898~75665796 | 4.28×10^-4^ | 9.62×10^-6^ | 5.57×10^-3^ | -0.049 |
| *GPR22* | 7 | 107010501~107216125 | 1.78×10^-4^ | 3.63×10^-4^ | 1.89×10^-3^ | 0.030 |
| *GPS2* | 17 | 7115977~7318658 | 6.90×10^-8^ | 3.11×10^-5^ | 2.52×10^-5^ | 0.149 |
| *GTPBP3* | 19 | 17345790~17553540 | 4.21×10^-4^ | 1.01×10^-4^ | 5.11×10^-4^ | -0.070 |
| *H19* | 11 | 1916405~2119065 | 1.44×10^-6^ | 1.09×10^-17^ | 3.65×10^-4^ | 0.121 |
| *HSPA4* | 5 | 132287661~132540709 | 1.36×10^-5^ | 3.18×10^-5^ | 4.51×10^-5^ | 0.147 |
| *KCTD11* | 17 | 7155207~7358262 | 2.10×10^-7^ | 1.03×10^-6^ | 4.72×10^-3^ | 0.122 |
| *KREMEN1* | 22 | 29369065~29664321 | 4.58×10^-6^ | 6.04×10^-6^ | 9.79×10^-3^ | -0.037 |
| *LAMTOR2* | 1 | 155924516~156128301 | 2.04×10^-5^ | 5.72×10^-6^ | 8.00×10^-3^ | 0.056 |
| *LEKR1* | 3 | 156444095~156863918 | 2.08×10^-11^ | 7.74×10^-4^ | 4.73×10^-4^ | -0.050 |
| *LOC100506457* | 2 | 12047241~12818474 | 3.03×10^-4^ | 3.98×10^-4^ | 8.60×10^-8^ | -0.033 |
| *LOC100652768* | 11 | 116966328~117172630 | 3.44×10^-4^ | 6.07×10^-5^ | 9.41×10^-3^ | -0.206 |
| *LOC102724927* | 16 | 3897625~4100445 | 2.38×10^-4^ | 7.20×10^-9^ | 2.66×10^-4^ | -0.022 |
| *LOC339539* | 1 | 43223292~43454463 | 9.13×10^-5^ | 3.48×10^-4^ | 3.63×10^-3^ | -0.097 |
| *MEX3A* | 1 | 155941803~156151789 | 8.08×10^-5^ | 5.67×10^-7^ | 1.79×10^-3^ | 0.057 |
| *MIR2392* | 14 | 101180827~101380911 | 7.40×10^-6^ | 1.52×10^-4^ | 7.84×10^-7^ | 0.139 |
| *MIR324* | 17 | 7026615~7226698 | 4.28×10^-10^ | 1.81×10^-4^ | 2.17×10^-4^ | -0.037 |
| *MIR493* | 14 | 101235396~101435485 | 4.46×10^-4^ | 2.27×10^-4^ | 8.01×10^-3^ | 0.147 |
| *MIR6738* | 1 | 155821063~156021127 | 4.75×10^-4^ | 4.05×10^-6^ | 1.93×10^-3^ | 0.078 |
| *MIR675* | 11 | 1917988~2118061 | 7.99×10^-7^ | 4.00×10^-15^ | 6.99×10^-3^ | 0.121 |
| *MIR770* | 14 | 101218726~101418824 | 4.34×10^-5^ | 7.18×10^-4^ | 3.94×10^-3^ | 0.161 |
| *MIR7851_1* | 1 | 155896957~156232001 | 3.31×10^-4^ | 2.98×10^-10^ | 5.57×10^-3^ | 0.058 |
| *MPI* | 15 | 75082351~75291798 | 4.86×10^-4^ | 7.62×10^-5^ | 1.89×10^-3^ | -0.107 |
| *MRPL11* | 11 | 66102549~66306310 | 6.44×10^-4^ | 5.84×10^-6^ | 2.52×10^-5^ | 0.078 |
| *MRPL23* | 11 | 1868501~2077839 | 1.25×10^-4^ | 1.16×10^-29^ | 5.11×10^-4^ | 0.155 |
| *MRPL23-AS1* | 11 | 1904438~2111150 | 2.66×10^-6^ | 1.10×10^-21^ | 3.65×10^-4^ | 0.129 |
| *MRPL34* | 19 | 17316476~17517652 | 8.08×10^-4^ | 6.77×10^-5^ | 4.51×10^-5^ | -0.026 |
| *NEURL4* | 17 | 7118950~7332638 | 4.69×10^-8^ | 9.91×10^-6^ | 4.72×10^-3^ | 0.171 |
| *NPAS4* | 11 | 66088474~66294177 | 5.20×10^-4^ | 2.59×10^-6^ | 9.79×10^-3^ | 0.054 |
| *OCIAD1* | 4 | 48733059~48963834 | 7.76×10^-5^ | 6.18×10^-4^ | 8.00×10^-3^ | -0.111 |
| *PAFAH1B2* | 11 | 116914999~117148889 | 4.89×10^-4^ | 3.56×10^-5^ | 4.73×10^-4^ | -0.200 |
| *PCSK7* | 11 | 116975786~117203241 | 4.08×10^-4^ | 1.26×10^-4^ | 8.60×10^-8^ | -0.160 |
| *PDE6C* | 10 | 95272344~95525429 | 4.13×10^-5^ | 7.77×10^-4^ | 9.41×10^-3^ | -0.094 |
| *PELI3* | 11 | 66133797~66344808 | 1.90×10^-4^ | 1.86×10^-6^ | 2.66×10^-4^ | 0.088 |
| *PHF23* | 17 | 7038346~7242825 | 2.54×10^-11^ | 1.64×10^-4^ | 3.63×10^-3^ | -0.035 |
| *RAB25* | 1 | 155930965~156140295 | 9.01×10^-5^ | 1.48×10^-6^ | 1.79×10^-3^ | 0.055 |
| *RBM14* | 11 | 66284052~66497397 | 3.87×10^-4^ | 8.31×10^-5^ | 7.84×10^-7^ | 0.201 |
| *RBM14-RBM4* | 11 | 66284052~66513944 | 3.99×10^-4^ | 3.08×10^-5^ | 2.17×10^-4^ | 0.196 |
| *SBF2* | 11 | 9700213~10415754 | 1.33×10^-4^ | 6.00×10^-6^ | 8.01×10^-3^ | -0.019 |
| *SCAMP2* | 15 | 75037196~75265670 | 6.73×10^-4^ | 7.42×10^-6^ | 1.93×10^-3^ | -0.021 |
| *SIDT2* | 11 | 116949938~117168161 | 4.53×10^-4^ | 2.16×10^-5^ | 6.99×10^-3^ | -0.206 |
| *SIK3* | 11 | 116614117~117069131 | 6.24×10^-5^ | 1.33×10^-4^ | 3.94×10^-3^ | -0.108 |
| *SLC2A1* | 1 | 43291045~43524847 | 5.84×10^-5^ | 5.09×10^-6^ | 5.57×10^-3^ | -0.055 |
| *SLC2A1-AS1* | 1 | 43324719~43549029 | 6.14×10^-5^ | 6.63×10^-5^ | 1.89×10^-3^ | -0.090 |
| *SLC2A4* | 17 | 7085053~7291367 | 4.47×10^-9^ | 1.84×10^-5^ | 2.52×10^-5^ | 0.033 |
| *SSR2* | 1 | 155878838~156090758 | 2.45×10^-5^ | 1.06×10^-6^ | 5.11×10^-4^ | 0.059 |
| *TAGLN* | 11 | 116970039~117175508 | 3.66×10^-4^ | 5.35×10^-5^ | 3.65×10^-4^ | -0.194 |
| *TMEM95* | 17 | 7158496~7360538 | 9.55×10^-7^ | 4.96×10^-7^ | 4.51×10^-5^ | 0.116 |
| *UBQLN4* | 1 | 155905091~156123516 | 1.12×10^-5^ | 6.45×10^-6^ | 4.72×10^-3^ | 0.050 |
| *YBX2* | 17 | 7091570~7297876 | 4.38×10^-9^ | 1.60×10^-5^ | 9.79×10^-3^ | 0.044 |
| *ZCCHC10* | 5 | 132232677~132462240 | 1.57×10^-5^ | 1.46×10^-5^ | 8.00×10^-3^ | 0.164 |
| *ZDHHC24* | 11 | 66206734~66413671 | 1.36×10^-4^ | 6.73×10^-5^ | 4.73×10^-4^ | 0.126 |

Note: the Pearson’s correction coefficient for SNP effect sizes of birthweight and breast cancer for a pleiotropic gene is shown in the last column.

## Table S5. Pleiotropic genes for maternal-specific birthweight and breast cancer detected by MAIUP

| gene | chr | start ~ stop | *P* (birthweight) | *P* (breast cancer) | FDR | Pearson |
| --- | --- | --- | --- | --- | --- | --- |
| *ANO8* | 19 | 17334031~17545638 | 2.95×10^-5^ | 5.61×10^-5^ | 1.29×10^-3^ | 0.095 |
| *BBS1* | 11 | 66178118~66401084 | 2.01×10^-4^ | 1.22×10^-5^ | 7.00×10^-3^ | -0.046 |
| *BLM* | 15 | 91160557~91458692 | 2.59×10^-6^ | 1.25×10^-4^ | 5.66×10^-3^ | 0.083 |
| *C15orf39* | 15 | 75394220~75604510 | 5.22×10^-8^ | 5.10×10^-5^ | 3.48×10^-3^ | 0.018 |
| *CCDC63* | 12 | 111184763~111445339 | 3.15×10^-4^ | 2.22×10^-6^ | 2.24×10^-4^ | 0.031 |
| *CDKAL1* | 6 | 20434687~21332634 | 8.92×10^-5^ | 1.07×10^-8^ | 7.00×10^-3^ | 0.009 |
| *COMMD4* | 15 | 75528336~75734268 | 7.66×10^-5^ | 4.40×10^-6^ | 3.40×10^-3^ | 0.168 |
| *COX5A* | 15 | 75112616~75330495 | 5.52×10^-10^ | 4.11×10^-4^ | 4.12×10^-3^ | 0.021 |
| *CPLX3* | 15 | 75018950~75224136 | 8.35×10^-9^ | 1.04×10^-5^ | 7.96×10^-3^ | 0.121 |
| *CSK* | 15 | 74974424~75195539 | 2.75×10^-8^ | 2.46×10^-5^ | 3.12×10^-3^ | 0.119 |
| *CUX2* | 12 | 111371827~111888358 | 2.80×10^-9^ | 2.14×10^-6^ | 6.26×10^-4^ | 0.021 |
| *CYP1A1* | 15 | 74911882~75117877 | 5.02×10^-6^ | 4.53×10^-4^ | 7.98×10^-5^ | 0.192 |
| *CYP1A2* | 15 | 74941183~75148941 | 1.52×10^-7^ | 2.20×10^-5^ | 1.45×10^-4^ | 0.136 |
| *DDA1* | 19 | 17320336~17534106 | 7.60×10^-5^ | 1.66×10^-4^ | 6.84×10^-5^ | 0.040 |
| *DPP3* | 11 | 66147483~66377130 | 2.85×10^-4^ | 8.15×10^-6^ | 9.50×10^-3^ | -0.009 |
| *EBF1* | 5 | 158022922~158626788 | 4.31×10^-7^ | 6.10×10^-24^ | 3.86×10^-4^ | 0.104 |
| *FAM219B* | 15 | 75092327~75299462 | 6.06×10^-10^ | 7.47×10^-5^ | 3.09×10^-3^ | 0.121 |
| *FES* | 15 | 91327664~91539006 | 5.37×10^-6^ | 1.46×10^-10^ | 5.29×10^-3^ | 0.106 |
| *FURIN* | 15 | 91311821~91526688 | 4.14×10^-5^ | 7.63×10^-9^ | 9.50×10^-3^ | 0.124 |
| *GOLGA6C* | 15 | 75450898~75665796 | 2.32×10^-7^ | 9.62×10^-6^ | 4.65×10^-3^ | 0.077 |
| *GOLGA6D* | 15 | 75475181~75688148 | 5.67×10^-6^ | 3.67×10^-7^ | 1.06×10^-4^ | 0.073 |
| *GTPBP3* | 19 | 17345790~17553540 | 5.23×10^-5^ | 1.01×10^-4^ | 2.24×10^-3^ | 0.098 |
| *HDDC3* | 15 | 91374147~91575799 | 1.00×10^-6^ | 2.66×10^-12^ | 1.88×10^-3^ | 0.109 |
| *LMAN1L* | 15 | 75005193~75218099 | 1.70×10^-8^ | 1.03×10^-5^ | 6.84×10^-5^ | 0.120 |
| *LOC100131138* | 12 | 111274405~111475250 | 5.34×10^-5^ | 6.38×10^-7^ | 2.23×10^-4^ | 0.021 |
| *LOC100652768* | 11 | 116966328~117172630 | 3.73×10^-4^ | 6.07×10^-5^ | 2.18×10^-3^ | 0.173 |
| *MAN2A2* | 15 | 91347419~91565815 | 4.41×10^-6^ | 2.11×10^-11^ | 6.84×10^-5^ | 0.092 |
| *MAN2C1* | 15 | 75548132~75760968 | 8.75×10^-5^ | 3.37×10^-6^ | 9.50×10^-3^ | 0.173 |
| *MIR4513* | 15 | 74981012~75181098 | 3.05×10^-8^ | 9.76×10^-6^ | 6.84×10^-5^ | 0.151 |
| *MIR631* | 15 | 75545951~75746026 | 9.68×10^-5^ | 4.98×10^-6^ | 2.33×10^-3^ | 0.154 |
| *MIR6882* | 15 | 75032982~75233048 | 5.88×10^-9^ | 9.51×10^-6^ | 2.23×10^-4^ | 0.102 |
| *MPI* | 15 | 75082351~75291798 | 1.13×10^-9^ | 7.62×10^-5^ | 3.47×10^-3^ | 0.119 |
| *MYL2* | 12 | 111248623~111458404 | 1.15×10^-4^ | 6.84×10^-7^ | 7.00×10^-3^ | 0.021 |
| *NEIL1* | 15 | 75539330~75747592 | 9.99×10^-5^ | 6.12×10^-6^ | 6.84×10^-5^ | 0.156 |
| *PCSK7* | 11 | 116975786~117203241 | 3.77×10^-5^ | 1.26×10^-4^ | 6.75×10^-3^ | 0.145 |
| *PELI3* | 11 | 66133797~66344808 | 2.74×10^-4^ | 1.86×10^-6^ | 3.70×10^-3^ | 0.000 |
| *PLVAP* | 19 | 17362263~17588137 | 2.27×10^-4^ | 2.35×10^-4^ | 6.84×10^-5^ | 0.072 |
| *PTPN9* | 15 | 75659461~75971632 | 2.53×10^-4^ | 1.75×10^-6^ | 6.84×10^-5^ | 0.164 |
| *RCCD1* | 15 | 91398105~91606355 | 4.38×10^-5^ | 5.48×10^-10^ | 4.60×10^-3^ | 0.109 |
| *SCAMP2* | 15 | 75037196~75265670 | 1.28×10^-9^ | 7.42×10^-6^ | 3.71×10^-3^ | 0.117 |
| *SIN3A* | 15 | 75561719~75848124 | 3.37×10^-4^ | 2.62×10^-6^ | 9.55×10^-3^ | 0.156 |
| *SST* | 3 | 187286693~187488201 | 3.06×10^-4^ | 3.70×10^-4^ | 7.54 ×10^-3^ | -0.076 |
| *TAGLN* | 11 | 116970039~117175508 | 3.78×10^-4^ | 5.35×10^-5^ | 9.39×10^-3^ | 0.168 |
| *TCF7L2* | 10 | 114610008~115027436 | 2.65×10^-5^ | 4.30×10^-14^ | 9.50×10^-3^ | 0.059 |
| *TET2* | 4 | 105967031~106300960 | 4.80×10^-6^ | 2.45×10^-5^ | 2.40×10^-3^ | 0.020 |
| *ULK3* | 15 | 75028456~75235687 | 3.43×10^-9^ | 1.12×10^-5^ | 9.50×10^-3^ | 0.118 |
| *UNC45A* | 15 | 91373409~91597323 | 1.12×10^-6^ | 2.16×10^-11^ | 6.84×10^-5^ | 0.103 |
| *ZBTB38* | 3 | 140943054~141268632 | 4.14×10^-6^ | 6.18×10^-14^ | 9.50×10^-3^ | 0.040 |
| *ZDHHC24* | 11 | 66206734~66413671 | 3.20×10^-4^ | 6.73×10^-5^ | 1.29×10^-3^ | -0.078 |

Note: the Pearson’s correction coefficient for SNP effect sizes of birthweight and breast cancer for a pleiotropic gene is shown in the last column.

## Table S6. Estimated effect sizes from the MR analysis for all the eight models

|  | *X*→*Y (Model 1)* | |  | *X*→*M*_1_ *(Model 2)* | |  | *X*→*M*_2_ *(Model 3)* | |  | *M*_1_→*M*_2_ *(Model 4)* | |
| --- | --- | --- | --- | --- | --- | --- | --- | --- | --- | --- | --- |
|  | OR (95% CIs) | *P* |  | OR (95% CIs) | *P* |  | OR (95% CIs) | *P* |  | OR (95% CIs) | *P* |
| *X*_1_ | 0.989 (0.902 ~ 1.083) | 0.806 |  | 1.093 (1.019 ~ 1.171) | 0.012 |  | 1.065 (0.790 ~ 1.434) | 0.681 |  | 0.852 (0.750 ~ 0.970) | 0.015 |
| *X*_2_ | 0.909 (0.775 ~ 1.067) | 0.244 |  | 0.994 (0.894 ~ 1.105) | 0.907 |  | 0.719 (0.487 ~ 1.064) | 0.099 |  |  |  |

|  | *X+M*_1_→*M*_2_ *(Model 5)* | |  | *X+M*_1_→*Y (Model 6)* | |  | *X+M*_2_→*Y (Model 7)* | | |
| --- | --- | --- | --- | --- | --- | --- | --- | --- | --- |
|  | OR (95% CIs) | *P* |  | OR (95% CIs) | *P* |  |  | OR (95% CIs) | *P* |
| *X*_1_ | 0.990 (0.790 ~ 1.240) | 0.927 |  | 1.010 (0.915 ~ 1.114) | 0.848 |  | *X*_1_ | 1.096 (0.956 ~ 1.257) | 0.190 |
| *M*_1_ | 1.119 (1.008 ~ 1.239) | 0.035 |  | 1.014 (0.971 ~ 1.060) | 0.525 |  | *M*_2_ | 1.036 (1.019 ~ 1.054) | 3.968×10^-5^ |
| *X*_2_ | 0.914 (0.659 ~ 1.268) | 0.590 |  | 0.906 (0.806 ~ 1.018) | 0.096 |  | *X*_2_ | 0.897 (0.775 ~ 1.038) | 0.145 |
| *M*_1_ | 1.131 (0.994 ~ 1.286) | 0.062 |  | 1.020 (0.976 ~ 1.066) | 0.368 |  | *M*_2_ | 1.033 (1.016 ~ 1.050) | 1.020×10^-4^ |

|  | *X+M*_1_*+M*_2_→*Y (Model 8)* | |
| --- | --- | --- |
|  | OR (95% CIs) | *P* |
| *X*_1_ | 1.089 (0.955 ~ 1.243) | 0.203 |
| *M*_1_ | 0.991 (0.931 ~ 1.055) | 0.780 |
| *M*_2_ | 1.020 (1.002 ~ 1.038) | 0.029 |
| *X*_2_ | 0.899 (0.783 ~ 1.032) | 0.130 |
| *M*_1_ | 0.999 (0.940 ~ 1.062) | 0.977 |
| *M*_2_ | 1.019 (1.002 ~ 1.036) | 0.032 |

Note: *X*_1_: fetal-specific birthweight; *X*_2_: maternal-specific birthweight; *M*_1_: age at menarche; *M*_2_: age at menopause; *Y*: breast cancer; OR: odds ratio; CIs: confidence intervals.

## Table S7. MR sensitivity analysis and pleiotropic test

| exposure | outcome | IVW | |  | MR-Egger test | |  | Weight median test | |  | Likelihood test | |  | MR-PRESSO | |
| --- | --- | --- | --- | --- | --- | --- | --- | --- | --- | --- | --- | --- | --- | --- | --- |
|  |  | OR  (95% CIs) | *P* |  | OR  (95% CIs) | *P* |  | OR  (95% CIs) | *P* |  | OR  (95% CIs) | *P* |  | RSSobs | *P* |
| *X*_1_ | *Y* | 0.989  (0.902 ~ 1.083) | 0.806 |  | 1.049  (0.828 ~ 1.329) | 0.685 |  | 0.999  (0.902 ~ 1.106) | 0.981 |  | 0.989  (0.899 ~ 1.086) | 0.812 |  | 144.019 | <0.001 |
| *X*_2_ | *Y* | 0.909  (0.775 ~ 1.067) | 0.244 |  | 1.084  (0.657 ~ 1.791) | 0.745 |  | 0.895  (0.792 ~ 1.011) | 0.076 |  | 0.901  (0.759~ 1.072) | 0.241 |  | 202.845 | <0.001 |
| *X*_1_ | *M*_1_ | 1.093  (1.019 ~ 1.171) | 0.012 |  | 1.213  (1.011 ~ 1.455) | 0.038 |  | 1.108  (1.024 ~ 1.199) | 0.010 |  | 1.097  (1.021 ~ 1.179) | 0.012 |  | 116.481 | 0.576 |
| *X*_2_ | *M*_1_ | 0.994  (0.894 ~ 1.105) | 0.907 |  | 1.186  (0.852 ~ 1.653) | 0.303 |  | 0.931  (0.847 ~ 1.023) | 0.139 |  | 0.993  (0.887 ~ 1.111) | 0.903 |  | 135.022 | <0.001 |
| *X*_1_ | *M*_2_ | 1.065  (0.790 ~ 1.434) | 0.681 |  | 0.945  (0.279 ~ 3.196) | 0.924 |  | 1.147  (0.727 ~ 1.809) | 0.557 |  | 1.067  (0.744 ~ 1.529) | 0.725 |  | 76.896 | 0.122 |
| *X*_2_ | *M*_2_ | 0.719  (0.487 ~ 1.064) | 0.099 |  | 1.169  (0.168 ~ 8.130) | 0.866 |  | 0.624  (0.359 ~ 1.084) | 0.094 |  | 0.727  (0.465 ~ 1.136) | 0.162 |  | 166.009 | <0.001 |
| *M*_1_ | *M*_2_ | 0.852  (0.750 ~ 0.970) | 0.015 |  | 1.111  (0.877 ~ 1.408) | 0.384 |  | - | - |  | 0.852  (0.748 ~ 0.971) | 0.016 |  | 112.386 | 0.773 |

Note: *X*_1_: fetal-specific birthweight; *X*_2_: maternal-specific birthweight; *M*_1_: age at menarche; *M*_2_: age at menopause; *Y*: breast cancer; OR: adds ratio; CIs: confidence intervals.

## Table S8. MR analysis after removing outlier indices with MR-PRESSO

| Exposure | Outcome | IVW | |  | MR-Egger test | |  | Weight median test | |  | Likelihood test | |
| --- | --- | --- | --- | --- | --- | --- | --- | --- | --- | --- | --- | --- |
|  |  | OR  (95% CIs) | *P* |  | OR  (95% CIs) | *P* |  | OR  (95% CIs) | *P* |  | OR  (95% CIs) | *P* |
| *X*_1_ | *Y* | 0.973  (0.895 ~ 1.057) | 0.514 |  | 1.030  (0.830 ~ 1.277) | 0.785 |  | 0.978  (0.884 ~ 1.082) | 0.665 |  | 0.971  (0.891 ~ 1.058) | 0.506 |
| *X*_2_ | *Y* | 0.957  (0.863 ~ 1.061) | 0.405 |  | 0.959  (0.703 ~ 1.309) | 0.788 |  | 0.898  (0.794 ~ 1.015) | 0.085 |  | 0.958  (0.862~ 1.065) | 0.424 |
| *X*_2_ | *M*_1_ | 1.019  (0.940 ~ 1.105) | 0.649 |  | 1.330  (1.050 ~ 1.684) | 0.020 |  | 0.955  (0.869 ~ 1.050) | 0.342 |  | 1.019  (0.938 ~ 1.108) | 0.644 |
| *X*_2_ | *M*_2_ | 0.828  (0.589 ~ 1.145) | 0.076 |  | 1.155  (0.892 ~ 6.400) | 0.673 |  | 0.777  (0.453 ~ 0.989) | 0.089 |  | 0.765  (0.546 ~ 1.116) | 0.112 |

Note: *X*_1_: fetal-specific birthweight; *X*_2_: maternal-specific birthweight; *M*_1_: age at menarche; *M*_2_: age at menopause; *Y*: breast cancer; OR: adds ratio; CIs: confidence intervals.

**
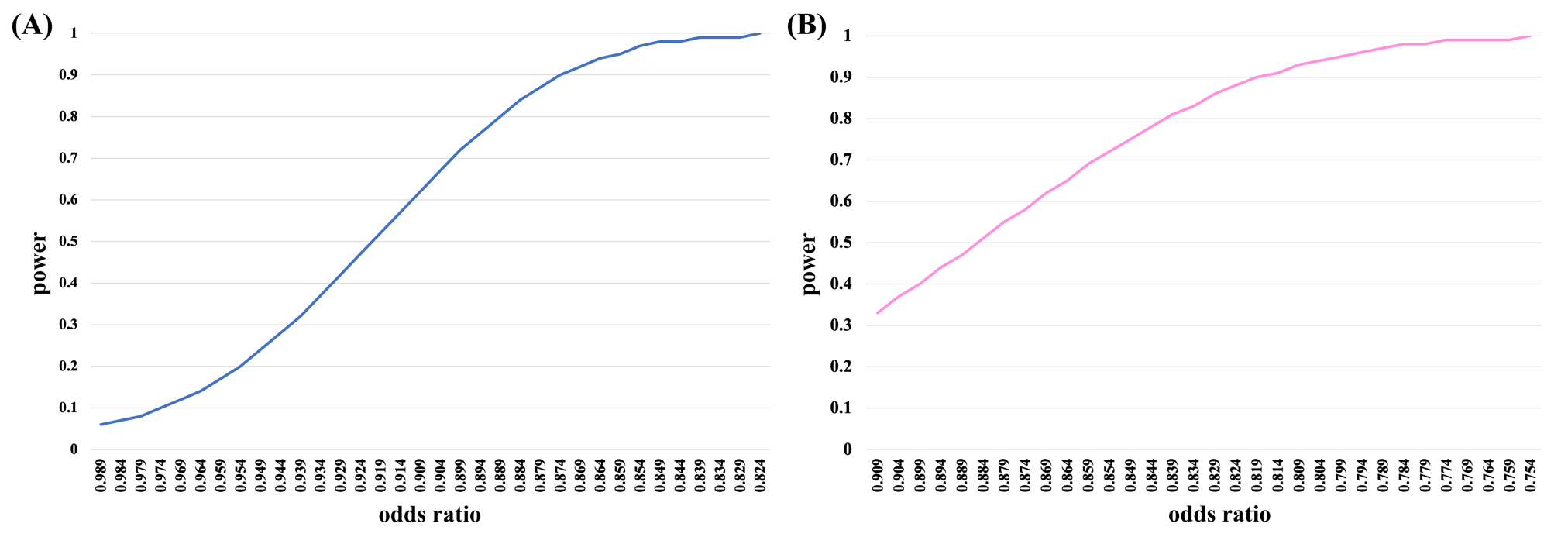
**

## Figure S1. Statistical power of MR analysis for fetal/maternal-specific birthweight and breast cancer when taking different ORs in simulation studies. (A) the power of fetal-specific birthweight and breast cancer; (B) the power of maternal-specific birthweight and breast cancer. Here, the proportion of variance explained by instruments was 0.011 for fetal-specific birthweight and 0.005 for maternal-specific birthweight; the proportion of breast cancer cases was 0.537 for both cases, and the significance level was set to 0.05.
